# Computational Biology and Bioinformatics in Nigeria

**DOI:** 10.1371/journal.pcbi.1003516

**Published:** 2014-04-24

**Authors:** Segun A. Fatumo, Moses P. Adoga, Opeolu O. Ojo, Olugbenga Oluwagbemi, Tolulope Adeoye, Itunuoluwa Ewejobi, Marion Adebiyi, Ezekiel Adebiyi, Clement Bewaji, Oyekanmi Nashiru

**Affiliations:** 1 H3Africa Bioinformatics Network (H3ABioNet) Node, National Biotechnology Development Agency (NABDA), Federal Ministry of Science and Technology (FMST), Abuja, Nigeria; 2 Human Genetics Department, Wellcome Trust Sanger Institute, Cambridge, United Kingdom; 3 International Health Research Group, Department of Public Health & Primary Care, University of Cambridge, Cambridge, United Kingdom; 4 Computational and Evolutionary Biology/Bioinformatics, Faculty of Life Sciences, University of Manchester, Manchester, United Kingdom; 5 Microbiology Unit, Department of Biological Sciences, Nasarawa State University, Keffi, Nigeria; 6 Centre for Molecular Biosciences, University of Ulster, Coleraine, United Kingdom; 7 Chevron Biotechnology Centre, Federal University of Technology, Yola, Nigeria; 8 Department of Computer and Information Sciences, Covenant University, Ota, Nigeria; 9 H3ABioNet Node, Covenant University Bioinformatics Research (CUBRe), Ota, Nigeria; 10 University of Hradec Kralove, Kralove, Czech Republic; 11 Department of Biochemistry, University of Ilorin, Ilorin, Nigeria; National Institutes of Health, United States of America

## Abstract

Over the past few decades, major advances in the field of molecular biology, coupled with advances in genomic technologies, have led to an explosive growth in the biological data generated by the scientific community. The critical need to process and analyze such a deluge of data and turn it into useful knowledge has caused bioinformatics to gain prominence and importance. Bioinformatics is an interdisciplinary research area that applies techniques, methodologies, and tools in computer and information science to solve biological problems. In Nigeria, bioinformatics has recently played a vital role in the advancement of biological sciences. As a developing country, the importance of bioinformatics is rapidly gaining acceptance, and bioinformatics groups comprised of biologists, computer scientists, and computer engineers are being constituted at Nigerian universities and research institutes. In this article, we present an overview of bioinformatics education and research in Nigeria. We also discuss professional societies and academic and research institutions that play central roles in advancing the discipline in Nigeria. Finally, we propose strategies that can bolster bioinformatics education and support from policy makers in Nigeria, with potential positive implications for other developing countries.

## Introduction: A Brief Overview of the State of Bioinformatics in Nigeria

The advent of high-throughput sequencing and computational approaches to biological data analysis means biology will never be the same again, and Nigeria is one of the developing countries making modest progress with respect to this. It is obvious that computing and sequencing are rapidly and drastically changing the face of biology. This has led the global scientific community to the relatively new disciplines of computational biology and bioinformatics. Needless to say, it is common knowledge that bioinformatics asks lots of questions that cut across all life sciences and turns the answers into knowledge for the benefit of scientists, pharmaceutical companies, and the public. For example, the World Wide Web has made it possible for a single public database of genome sequence data to provide services through a uniform interface to a worldwide community of users [1].

In this article, our working definition of bioinformatics shall be “an emerging scientific field involving the development and integration of techniques, such as applied mathematics, statistics, computer science, chemistry, and biochemistry, to solve biological problems.” Obviously, by providing algorithms, databases, user interfaces, and statistical tools, bioinformatics makes it possible to complete important work such as comparative genomics, proteomics, interactomics, metabolomics, and all other “-omics” research. Over the years, there have been several bioinformatics training events, including workshops, and modest research in this field in Nigeria.

## Computational Biology and Bioinformatics in Nigeria: When It All Began

The beginning of bioinformatics in Nigeria dates back to the early part of year 2000, and so far, it is still developing, with modest accomplishments. To the best of our knowledge, four key organizations were initially involved in the development of bioinformatics in Nigeria. They include Covenant University, University of Ibadan, National Biotechnology Development Agency (NABDA), and University of Ilorin. These institutions organized several workshops, seminars, and symposia on bioinformatics at the beginning of the study of bioinformatics in Nigeria. In addition, the West African Biotechnology Workshops Series (WABWS) [2] pioneered efforts to provide training in bioinformatics to Nigerian scientists. Some of these initial awareness campaigns are discussed in subsequent sections.

## Bioinformatics Training and Education in Nigeria: The Beginning

The credit for the first national bioinformatics training awareness in Nigeria can go to the region-wide training courses on molecular biology and bioinformatics organized by the West African Biotechnology Workshops Series between 2002 and 2005. The series featured notable workshops, such as the Applied Malaria Bioinformatics Workshop in 2004, the WABWS Advanced HIV Sequence Analysis in 2005, and the WABWS Advanced Viral Bioinformatics Course in 2005, at the University of Ibadan.

The Covenant University's Computer and Information Science Department launched its bioinformatics activities with a workshop in 2005, which was tagged, “International Workshop on Pattern Discovery in Biology (IWPDB).” In 2009, Covenant University organized the second International Workshop on Pattern Discovery in Biology. This workshop was supported by EMBnet and focused on bridging the gap between biologists and bioinformaticians. The workshop highlighted the fundamentals of bioinformatics and the systematic approach for understanding biological data using computational methods [3]. This workshop attracted both local and foreign participants, who learnt the basics of bioinformatics and its applicability to biological sciences. Topics covered during the workshop included the design and analysis of algorithms, gene networks, gene expression, protein structure, genome comparisons, molecular sequence analysis, structural and functional genomics, microarray design and data analysis, combinatorial libraries and drug design, and recognition of genes and regulatory elements [3].

In furthering the development of the discipline in Nigeria, the University of Ilorin, through its Department of Physiology and Biochemistry, in collaboration with the West African Bioinformatics Research Institute (WABRI, established in 2003, now a private initiative), organized an introductory bioinformatics training workshop in 2006 and organized training in bioinformatics and computational molecular biology techniques in 2008. Since 2009, several intermediate and advanced bioinformatics workshops and/or courses have been held in different parts of Nigeria. For instance, Chevron Biotechnology Centre, Federal University of Technology, Yola, organized a series of hands-on training workshop on molecular biology and biotechnology between 2009 and 2011. These workshops featured significant training on bioinformatics. These training opportunities provided capacity building in bioinformatics for several Nigerian scientists and other scientists from neighboring countries.

However, although bioinformatics is taught at the postgraduate level in many Nigerian institutions, it remains the case that only a few Nigerian universities teach the course at the undergraduate level. Therefore, more in terms of bioinformatics research and education is expected from Nigeria. Nevertheless, some universities are making modest progress with respect to this. Worthy of note are the Covenant University, University of Ibadan, and University of Ilorin, which offer MSc and PhD degrees in computer science with specialization in bioinformatics. Other universities, such as the Federal University of Technology, Yola, offer bioinformatics as a course in their biotechnology and biochemistry degree programs.

## Bioinformatics Research in Nigeria

**Figure 1:**
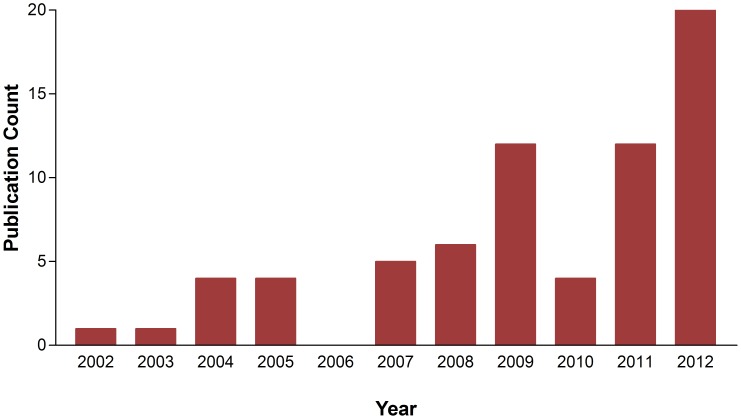
Bioinformatics-related publications per year by Nigerian scientists. The search terms “bioinformatics Nigeria,” “in silico Nigeria,” “computational biology Nigeria,” and “next generation sequencing Nigeria” were used to retrieve publications from the PubMed, Web of Science, and Scopus databases over the period of 2002 to 2012.

The current strength of bioinformatics and computational biology research work in Nigeria is not yet at its full potential because of inadequate institutional support. This notwithstanding, a new generation of Nigerian scientists with expertise in bioinformatics is emerging, and bioinformatics training is spreading. Therefore, it is hoped that bioinformatics research will receive a boost in the near future. As an indication of current and past research efforts in bioinformatics in Nigeria, Figure 1 shows bioinformatics-related publications by Nigerian scientists. The search terms “bioinformatics Nigeria,” “in silico Nigeria,” “computational biology Nigeria,” and “next generation sequencing Nigeria” were used to retrieve publications from the PubMed, Web of Science, and Scopus databases over the period of 2002 to 2012. These were then sorted for duplicate copies. Sixty nine publications were retrieved. Results presented in Figure 1 exclude some publications on bioinformatics in Nigeria [4]–[12], which were not indexed in the PubMed, Web of Science, and Scopus databases. In addition, the search terms used might have missed some relevant publications indexed in the databases [13]–[14].

The trend shows a consistent increase in bioinformatics publications in the last two years, with most of the bioinformatics research focusing mainly on malaria [15]–[41], a vector-borne disease common in the tropics. *Plasmodium* sp., the parasite that causes malaria, is transmitted by the female *Anopheles* mosquito. Resistance to anti-malarial drugs has become a major challenge, especially in Africa, where about 90% of malaria cases occur. This, in part, informs the research interest in this area. Covenant University's bioinformatics research cluster is one of the most active research clusters in the country. The group is currently leading bioinformatics research in malaria within the country. Other research foci of the cluster include studies involving microarrays and data mining/analysis, as well as database design and management.

Despite this progress in the field of malaria research in Nigeria, a lot still needs to be done and published in reputable journals that are indexed in leading databases. Searches from the PubMed, Web of Science, and Scopus databases retrieved only a limited number of publications from Nigerian scientists. In the same manner, NABDA is leading bioinformatics research in the country in the areas of human genome variation and visual analytics, in collaboration with the Wellcome Trust Sanger Institute (WTSI) and the Visual Analytics in Biology Curriculum Network, United States, respectively. WTSI has particularly played a major role in providing continuous research capacity building in Nigeria and other parts of Africa. Details of major research foci, as well as research projects in some bioinformatics research groups and academic institutions in Nigeria, are summarized in Table 1.

**Table 1:** Research foci and projects of bioinformatics research groups in Nigeria.

S/No	Research Group	Major Research Focus	Research Projects	Website
1	Covenant University Bioinformatics Research Group (CUBRe)	Malaria Drug Targets Identification and Validation, Microarrays	Bioinformatics Web Application (a) Development and implementation of a bioinformatics online distance education learning tool for Africa [1].	http://www.covenantuniversity.edu.ng, Accessed 7 January 2014
			Computational Model (a) *AnoSpEx*: A stochastic, spatially explicit computational model for studying *Anopheles* metapopulation dynamics [26]. (b) A stochastic computational model for *Anopheles* metapopulation dynamics: towards malaria control and insight for possible eradication [27].	
			Drug Resistance (a) In silico models for drug resistance [18]. (b) Computational discovery of drug resistance mechanism(s) of the malaria parasite to tetracyclines and chloroquines [23]. (c) In silico studies of multidrug resistance genetic markers of *Plasmodium* species [44].	
			Metabolic Networks/Drug Targets (a) Comparing metabolic network models based on genomic and automatically inferred enzyme information from *Plasmodium* and its human host to define drug targets in silico [18],[19]. (b) Estimating novel potential drug targets of *P. falciparum* by analyzing the metabolic network of knock-out strains in silico [20]. (c) In silico evaluation of malaria drug targets [28]. (d) Computational and experimental analysis identified 6-diazo-5-oxonorleucine as a potential agent for treating infection by *P. falciparum* [29]. (e) In silico detection of chokepoints enzymes in four plasmodium species [45].	
			Mobile Computing/Computational Biology MACbenabim: A multiplatform mobile application for searching key terms in computational biology and bioinformatics [30].	
			Databases/Bioinformatics (a) Development of a prototype hybrid grid-based computing framework for accessing bioinformatics databases and resources [31]. (b) Development of a secured information system to manage malaria-related cases in southwestern region of Nigeria [34]. (c) Repository of malaria drugs and insecticides resistance [16].	
			Expert System (a) Building a computer-based expert system for malaria environmental diagnosis: an alternative malaria control strategy [35]. (b) Implementation of XpertMalTyph: an expert system for medical diagnosis of the complications of malaria and typhoid [32]. (c) National Hospital Management Portal (NHMP); a framework for e-health implementation [33].	
			Genomics (a) The genomic landscape - How does *Plasmodium* compare to other apicomplexan species? [15] (b) Evaluating the relationship between a generic-based filtering program and DNA sequences [36]. (c) Clustering *P. falciparum* genes to their functional roles using k-means [24]. (e) New insights into the genetic regulation of *P. falciparum* obtained by Bayesian modelling [37]. (d) Detection of recombination in variable tandem repeats sequences [38].	
			Signaling Pathway (a) Computational identification of signaling pathways in *P. falciparum* [38].	
			Microarray/Bioinformatics (a) A comparative analysis of existing oligonucleotides selection algorithms for microarray technology [40]. (b) Experimental and computational applications of microarray technology for malaria eradication in Africa [41].	
2	National Biotechnology Development Agency(NABDA)	Microbiome, Human Genetics, Genome Variation, Visual Analytics	(a) Human genomic variation in cardiometabolic traits (in collaboration with WTSI Cambridge). (b) Quality control for NGS human genome data, read alignment and the calling of genetic variants from raw sequencing data. (c) Training and capacity development in bioinformatics analysis of human genomes in Africa component. (d) Making meaning of the Yoruba Genome (YRI) (e) Visual analytics of human genome variation datasets.	http://www.nabda.gov.ng, Accessed 7 January 2014
3	University of Ibadan	Machine Learning, Visual Analytics, Phylogenetic Inference		http://bioinformatics.ui.edu.ng, Accessed 7 January 2014
4	University of Lagos	Genetics, Cell and Molecular Biology, Biotechnology, Molecular Systematics and Bioinformatics, Ecology and Environmental Biology		http://cbg.unilag.edu.ng, Accessed 7 January 2014
5	University of Port-Harcourt	Biotechnology		http://www.uniport.edu.ng, Accessed 7 January 2014
6	Chevron Biotechnology Centre, Federal University of Technology, Yola	Biotechnology, Drug Discovery	(a) In silico design and modeling of peptide-based agents for treatment of noncommunicable diseases. (b) Gene and protein expression profiling in Type 2 diabetes and cancer. (c) Identification of targets of novel peptide-based antidiabetic drugs.	http://www.mautech.edu.ng, Accessed 7 January 2014
7	University of Benin	Biotechnology, Cell Biology and Genetics, Plant Biosystematics, Physiology and Biochemistry		http://www.uniben.edu/departments/plant-biology-and-biotechnology, Accessed 7 January 2014
8	Fountain University	Genetics and Biotechnology		http://www.fountainuniversity.edu.ng/index.php/styles/style2, Accessed 7 January 2014
9	Bells University	Biomedical Engineering, Biotechnology		http://www.bellsuniversity.org, Accessed 10 November 2013
10	Babcock University	Molecular Biology and Biotechnology, Biosystematics and Evolution, Cytogenetic and Genetic Engineering		http://www.babcockuni.edu.ng/main/index.php?option=com_content&view=article&id=166&Itemid=170, Accessed 10 November 2013
11	Wesley University of Science and Technology	Applied Biology and Biotechnology, Computational Analysis		http://www.wusto.edu.ng/pages/CNAS/CNAS%20page2.html, Accessed 10 December 2013
12	Caleb University	Physics with Computational Modeling, Industrial Biotechnology		http://calebuniversity.edu.ng/pages.php?id=52&parentid=65, Accessed 31 March 2014
13	Madonna University	Biomedical Engineering Technology		http://www.madonna.edu/academics/academic-programs/asx, Accessed 10 December 2013
14	Enugu State University of Science and Technology	Biotechnology, Genetics, Public Health and Parasitology, Genetics, Biophysics and Cytology		http://www.esut.edu.ng/cms-page.php?id=347, Accessed 10 November 2013
15	Federal University of Technology, Owerri	Cell, Tissue, and Enzyme Engineering; Medical and Veterinary Biotechnology; Renewable resources Technology, Environmental Biotechnology		http://www.futo.edu.ng/Schools/Science/biotechnology.aspx, Accessed 11 December 2013
16	Redeemer's University	Applied Biology and Genetics, Environmental, Biochemistry/Biotechnology		http://www.run.edu.ng/index.php?option=com_content&view=article&id=52&Itemid=135, Accessed 7 January 2014
17	University of Ibadan	Industrial Microbiology and Biotechnology		http://sci.ui.edu.ng/acadmcb, Accessed 7 January 2014
18	University of Jos Centre for Biotechnology and Genetic Engineering	Artemisia Cultivation for Malaria Therapy, Malaria Bioinformatics		http://www.channelstv.com/home/2012/02/23/unijos-cultivates-malaria-treatment-plant/, Accessed 7 January 2014
19	Institute of Human Virology, Abuja	Development of H3Africa Bio-Repository		www.ihvnigeria.org, Accessed 7 January 2014
20	Joseph Ayo Babalola University	Bioinformatics of Lassa Virus Glycoprotein		http://www.jabu.edu.ng/y/index.php?option=com_search&searchword=bioinformatics, Accessed 7 January 2014

In addition, there has been an upsurge of interest among Nigerian scientists in undertaking bioinformatics and computational biology research in institutions overseas. This has led to many of them pursuing such interests either as research fellows, doctoral students, or postdoctoral fellows in several institutions in Europe and the Americas. Expectedly, this is fostering opportunities for mutually beneficial international research collaborations and expanding the research networks of Nigerian scientists. It is hoped that this new attitude and interest will, in the near future, positively impact on the overall development of bioinformatics research and education in Nigeria.

## Institutions Currently Involved in Computational Biology and Bioinformatics in Nigeria

The number of institutions involved in bioinformatics education and research in Nigeria is gradually increasing at an encouraging pace. As a field of science that is relatively new in Nigeria, only a few institutions are currently actively involved in bioinformatics research in the country (Table 1). However, the number of institutions involved in bioinformatics education in Nigeria is gradually increasing at an encouraging pace. (For author information, see Box 1.)

**Box 1. Authors' Biographies**

**Segun A. Fatumo** was Founder and pioneer President of the Regional Student Group Africa (2007–2009) of the Student Council of the International Society for Computational Biology (ISCBSC). He is currently an H3ABioNet NABDA Node Visiting Research Fellow at the Wellcome Trust Sanger Institute and University of Cambridge. He is Vice President of Africa Society for Bioinformatics and Computational Biology (2011–present).

**Moses P. Adoga** is president of Regional Student Group West Africa (RSG West Africa) of the ISCB-SC and a faculty member at Nasarawa State University, Nigeria.

**Opeolu O. Ojo** is Research and Training Director at Chevron Biotechnology Centre, Modibbo Adama University of Technology, Yola, Nigeria and currently a Research Fellow at the University of Ulster, Coleraine, United Kingdom.

**Olugbenga Oluwagbemi** is Vice President of RSG West Africa and holds a PhD from Covenant University and formerly a Fulbright Research Fellow at the John Hopkins University, Baltimore, Maryland, US.

**Esther T. Adeoye** is currently a master's student of information management at the University of Hradec Kralove, Czech Republic and formerly a graduate research intern at Covenant University Bioinformatics Research Group.

**Itunuoluwa Ewejobi** is a faculty member and a PhD student at Covenant University and a former Research Fellow at the German Cancer Research Center (DKFZ), Heidelberg, Germany under the DAAD fellowship.

**Marion Adebiyi** is the immediate former President of RSG West Africa and a faculty member at Covenant University, Ota, Nigeria.

**Ezekiel F. Adebiyi** is Professor and Head of the Covenant University Bioinformatics Research (CUBRe) and Covenant University H3ABioNet Node.

**Clement O. Bewaji** is Professor of Biochemistry at the University of Ilorin, Nigeria and the Director of Research at WABRI.

**Oyekanmi Nashiru** is Professor and Director of molecular biology and bioinformatics department, National Biotechnology Development Agency, NABDA/FMST, Umar Musa Yar'A dua Expressway, Abuja, and H3ABioNet NABDA Node, Abuja, Nigeria.

## Participation in International Conferences

Bioinformatics research efforts in Nigeria have been thriving over the years with increased support from international scientific organizations for training, research, and conference participation. Many Nigerian members of the International Society for Computational Biology (ISCB) have benefitted from such support. This has made it possible for the members to present their work at conferences in different aspects of bioinformatics, biotechnology, and computational biology.

Within the African continent, many Nigerian members of the African Society for Bioinformatics and Computational Biology (ASBCB) hold prominent positions and play influential roles in the society. In the past few years, Nigeria produced the president of the society. Presently, Nigerians hold the position of vice president and secretary of the society. Many Nigerian scientists have participated significantly in organizing ISCB Africa and ASBCB conferences across the continent. For instance, in 2009, Nigerian bioinformatics students and scientists initiated and participated in the first African Virtual Conference [42]. This event was supported by the Bioinformatics Organization (www.bioinformatics.org), Regional Student Group Africa (RSG Africa), Regional Student Group Morocco (RSG Morocco), International Society for Computational Biology Student Council (ISCBSC), and ASBCB.

## Nigerian Society for Bioinformatics and Computational Biology (NiSBCB)

In 2005, at the first IWPDB, which took place at Covenant University, Ota, Nigeria, the Nigerian Society for Bioinformatics and Computational Biology (NiSBCB) was established. The society aims to strengthen and expand bioinformatics education and research across Nigeria through:

1. Promoting the exchange of ideas and resources in the fields of bioinformatics and computational biology and facilitating local and international collaborations among Nigerian scientists and educators.
2. Promoting the establishment of infrastructural facilities.
3. Facilitating access to bioinformatics and computational biology infrastructure.
4. Advancing and promoting bioinformatics and computational biology in Nigeria.
5. Serving a global community by influencing governmental and scientific policies, providing high quality research publications, and hosting professional meetings, and through distribution of valuable information about training, education, employment, and relevant news from related fields.
6. Developing the application of bioinformatics in Nigeria in collaboration with individuals, groups, and organizations.

## Nigerian Bioinformatics Research and Education Network (NBREN)

Due to the need to introduce bioinformatics to more universities in Nigeria [43], at a recent event of Bioinformatics Curriculum Development Workshop held at the University of Ibadan July 22–25, 2013, Nigerian Bioinformatics Research and Education Network (NBREN) was formed to further strengthen efforts towards bioinformatics development and research in Nigeria. Specific aims of the network include to:

1. Connect Nigerian researchers, educators, industrialists, and policy makers to resources and opportunities in the field of bioinformatics.
2. Showcase the benefits of bioinformatics for the development of the economy of Nigeria.
3. Promote the integration of bioinformatics in the Nigerian educational system.
4. Organize activities that promote bioinformatics through short courses, workshops, and seminars.
5. Link NBREN members with other bioinformatics networks and societies across the globe.

In addition, NBREN will organize bioinformatics-related events, facilitate research, recommend policies to the government, develop a national bioinformatics curriculum, and organize bioinformatics training workshops and conferences in Nigeria.

## Challenges of Bioinformatics in Nigeria

Challenges facing the introduction and the development of bioinformatics in Nigeria have been reviewed in earlier publications [2],[43]. Among other challenges, it has been highlighted that bioinformatics research in Nigeria urgently needs adequate funding from both private and public sectors. The majority of funding received so far for bioinformatics-related research has been sourced from international organizations, with little support from private bodies.

In addition to funding, other recognized challenges include inadequate facilities for consistent research, inadequate training and capacity-building opportunities, infrastructural problems such as inadequate power supply, and lack of appropriate national policy/strategy for bioinformatics education and research.

## Conclusion

The contribution of bioinformatics to developments in biological science education and research has been identified. Interestingly, as the awareness of the discipline is becoming more prevalent within the academia in Nigeria, so is the interest of new-generation scientists in this emerging scientific discipline. It is also clear that research in bioinformatics is gradually becoming entrenched in Nigeria and is becoming increasingly useful in tackling some of the health challenges facing the country and the rest of Africa.

However, the development of the discipline is faced with several challenges, ranging from funding to inadequate infrastructure. Public-sector–driven investment in scientific research and development, particularly bioinformatics, in Nigeria is minimal. Like in most developing countries, this has led home-based Nigerian scientists to rely on personal efforts in bioinformatics education and research. To a large extent, these efforts have been fruitful. The number of research articles in bioinformatics from Nigeria is increasing. Nigerian scientists are becoming more involved at both local and international levels. The teaching of the discipline at undergraduate and postgraduate levels within the nation is becoming more widespread. We therefore believe that bioinformatics and computational biology can be bolstered in Nigeria by the formulation and implementation of governmental policies that aim to foster the twin disciplines through research funding, capacity building, and pedagogic activities within universities and research institutions across the country.

**Websites and Links**

http://www.covenantuniversity.edu.ng

http://cubre.covenantuniversity.edu.ng

http://cubre.covenantuniversity.edu.ng/index.php/nigeria-society-for-bioinformatics-and-computatational-biology-nisbcb/

http://www.nabda.gov.ng/

http://www.run.edu.ng

http://www.uniben.edu

http://www.wabri.org

http://www.unilorin.edu.ng

http://www.asopah.org

http://public.tableausoftware.com/views/ngr_bioinfo/research_groups
